# Sevoflurane-Associated Acute Liver Injury in Renal Transplantation and Review of Literature

**DOI:** 10.1155/cria/1303993

**Published:** 2025-03-18

**Authors:** Imran Gani, Nawal Moin, Jeffery Fallah, Ahmad Mirza

**Affiliations:** ^1^Department of Nephrology, Hypertension and Transplant Medicine, Augusta University Medical Center, Medical College of Georgia, 1120 15th St, Augusta 30912, Georgia, USA; ^2^Department of Surgery, Augusta University Medical Center, Medical College of Georgia, 1120 15th St, Augusta 30912, Georgia, USA

**Keywords:** drug-induced acute liver injury, general anesthesia, halogenated hydrocarbons, immunosuppression, kidney transplantation, sevoflurane

## Abstract

Acute liver injury can be precipitated by several factors perioperatively. One of the rare factors identified intraoperatively is the use of sevoflurane, an inhalational anesthetic agent which can cause significant acute hepatotoxicity. The report presents a case of acute liver injury followed by graft loss in a patient who underwent kidney transplantation. The patient developed several complications which resulted in graft loss. Close postoperative monitoring of patients following kidney transplantation is crucial. The case supports the current literature describing sevoflurane as a hepatotoxic agent. Medication side effects should be closely monitored both intraoperatively and postoperatively in those with renal dysfunction.

## 1. Introduction

Inhaled halogenated compounds are widely used induction and maintenance anesthetic agents that were developed in the 1950s [1]. In contrast to diethyl ether and chloroform, the conventional and now historic anesthetics, halogenated anesthetics are non-flammable and boast a wider margin of safety, which contributed to their wider acceptance in clinical practice. Inhaled halogenated gases are efficacious in induction and can be easily delivered through vaporization [1]. Despite the significant improvement from their predecessors, there remain numerous safety profile concerns of halogenated anesthetics including hepatic toxicity and malignant hyperthermia [2, 3].

Hepatotoxicity is a common side effect of halogenated anesthetics, most classically associated with halothane [2]. The current proposed mechanism of hepatic injury is through CYP2E1-mediated production of trifluoroacetic acid, an immunologically active metabolite, that induces a hypersensitivity reaction, leading to subsequent hepatic damage. Trifluoroacetic acid binds to several liver proteins and becomes a neoantigen that precipitates the hepatotoxicity associated with halothane, especially after repeated exposures [2]. Given halothane's profound ability to induce severe hepatotoxicity, which limited its scope of clinical utility, additional halogenated anesthetics including isoflurane, desflurane, and sevoflurane were developed to improve the class prototype [1]. Of the halogenated anesthetic class, sevoflurane is generally considered to have the lowest risk of hepatotoxicity with many sources describing it as a safe alternative to other halogenated anesthetics [3, 4]. This assumption is based on research showing that sevoflurane had minimal metabolism by CYP2E1 as well as a scarcity of post-release data indicating apparent hepatic dysfunction following sevoflurane administration [2]. Despite this, multiple case reports have described hepatotoxicity associated with sevoflurane, which indicates that sevoflurane may not be as hepatic safe as once thought [5–10]. In this report, we describe a case of sevoflurane-associated liver injury following its intraoperative use for the duration of a kidney transplantation surgery that precipitated the subsequent loss of the donor kidney.

## 2. Clinical Case Presentation

A 70-year-old patient with a past medical history of hypertension and end-stage renal disease (ESRD) secondary to a congenitally malformed nonfunctional right kidney presented to our tertiary care center for kidney transplantation with a HLA–matched, blood group compatible (blood group B positive) deceased donor kidney. An extensive preoperative evaluation was performed including a hepatitis panel, human immunodeficiency virus (HIV), cytomegalovirus (CMV), and Epstein–Barr virus (EBV), which were all negative. The workup also included the cardiac workup with the electrocardiogram and echocardiogram. The virtual cross-match (computed generated algorithm to identify preformed antibodies in the recipient versus the donor antigen profile) and physical cross-match (mixing of the recipient and donor blood to identify hemolysis resulting from preformed antibodies) were both performed prior to transplantation. Patient's preoperative AST (14 U/L), ALT (9 U/L), bilirubin (0.7 mg/dL), albumin (3.6 g/dL), PT (10.6 s), PTT (29.1 s), INR (0.9), and fibrinogen (280 mg/dL) were in the normal range. Following an unremarkable preoperative evaluation, the patient was taken for kidney transplant surgery.

Prior to incision, immunosuppressant induction therapy with methylprednisolone (500 mg) and basiliximab (20 mg) was administered intravenously. The anesthetic regimen included induction with propofol (1 mg/kg), fentanyl (2 mcg/kg), and rocuronium (0.6 mg/kg). Maintenance anesthesia was performed with sevoflurane (1.7% in oxygen) for the duration of the operation. The kidney transplant operation lasted four hours, without intraoperative complications with minimal blood loss. There was no requirement for vasopressors during the transplant. The patient's heart rate varied in the range of 68–86 beats/min and systolic BP was maintained in the range of 110–160 mmHg. Prior to closure, the donor's kidney appeared pink and well-perfused with a robust doppler signal. The patient's vitals were stable throughout the surgery.

Five hours postoperatively, the patient became increasingly somnolent. The patient's Glasgow coma score was reported as 12 (eye = 3, verbal = 4, and motor = 5). The patient's abdominal examination showed mild tenderness in the right iliac fossa at the incision site and the rest of the abdominal examination was unremarkable. An arterial blood gas (ABG) revealed a marked metabolic acidosis with a pH of 6.8 (reference range: 7.31–7.42), serum bicarbonate of 4 mmol/L (ref: 22.0–26.0 mmol/L), and lactic acid of 10 mmol/L (normal: < 2 mmol/L) (Table 1). Given the severity of acidosis, a bicarbonate bolus (50 mEq) was administered and later transitioned to an intravenous infusion (2 mEq/kg). Additional laboratory evaluation revealed findings consistent with acute liver injury (ALI) with significant transaminitis (AST 2673 U/L), ALT (2680 U/L), hyperammonemia (137 mcg/dL), prolonged PT (25.4 s), prolonged PTT (41.4 s), and elevated INR (2.2) (Table 1). The patient was transferred to the surgical intensive care unit. She also underwent urgent hemodialysis for correction of acidosis.

The patient's clinical condition continued to worsen the following day as 1100 mL of serosanguineous drain output was noted from the surgical site with a four-point decrease in hemoglobin. Multiple units of packed red blood cells (pRBCs) were transfused and the patient was taken to the OR for an emergency exploratory laparotomy to evaluate for intraabdominal hemorrhage. Intraoperatively, the transplanted kidney appeared grossly edematous and congested with diffuse oozing from the surgical bed. Additional units of pRBCs, fresh frozen plasma, and desmopressin were administered intraoperatively for coagulopathy. Flow doppler signals were present at both the arterial and venous end of the transplanted kidney and in the cortex of the kidney. Given clinically evident coagulopathy and tissue friability, the surgical wound was left open with temporary closure with gauze and abdominal binder. The allograft function did not improve, and the patient continued to be dialysis-dependent.

Two days later, the patient returned to the operating room to re-evaluate the transplanted kidney and to achieve permanent closure of the surgical site. Intraoperatively, the donor's kidney appeared dusky and engorged with worsening edema. At this time, Doppler signals were absent throughout the donor's kidney. However, the doppler signals were still intact at both the arterial and venous anastomosis. The nonviable kidney was explanted, and the wound was closed primarily. We performed a repeat physical cross-match of the donor with the recipient serum and the result was negative. Donor-specific antibody testing was negative as well. Histopathology of the explanted kidney showed infraction of the transplanted kidney.

**Figure 1:**
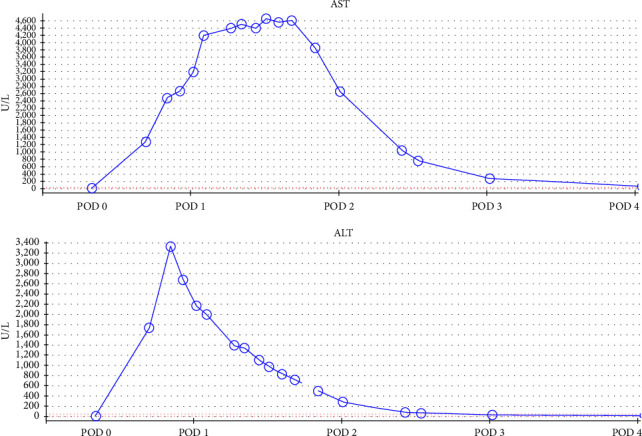
Aspartate aminotransferase (AST) and alanine aminotransferase (ALT) trend over the four postoperative (POD) days.

Coagulopathy gradually improved with complete clinical resolution by postoperative Day 4, alongside continuous laboratory improvement (Table 1). Similarly, hepatic enzymes improved with the downtrending of AST and ALT with complete improvement as seen in Table 1 and Figure 1. The remainder of the postoperative course was complicated by wound hematoma that required drainage and fascia-only closure with subsequent wound vac placement. Following a 17-day length of stay, the patient was discharged to a rehabilitation center in stable condition and on maintenance hemodialysis.

## 3. Discussion

ALI is a potentially life-threatening alteration in hepatic function. The presentation of ALI varies and is based on the etiology and severity of hepatic dysfunction. ALI can cause potentially fulminant hepatic failure with the development of coagulopathy, encephalopathy, and evidence of hepatic dysfunction on laboratory evaluation [11–13]. On laboratory evaluation, elevations in AST, ALT, INR, and ALP indicate hepatic dysfunction. A ratio of ALT to ALP greater than five indicates a hepatocellular pattern of damage and is helpful in differentiating patterns of liver injury [14].

Managing patients with ALI requires intensive supportive care to manage resultant metabolic abnormalities, coagulopathy, and cerebral edema [12]. Metabolic derangements in ALI are potentially life-threatening as severe pH disturbances, electrolyte abnormalities, and profound hypoglycemia can occur. Our patient developed severe metabolic acidosis which required bicarbonate infusion and urgent hemodialysis. Coagulopathy is especially problematic for patients in the postoperative period and should be managed with the replacement of blood components including pRBCs, fresh frozen plasma or cryoprecipitate, and platelet transfusions [12]. Depending on the severity of the liver injury which leads to liver failure, liver transplantation may be required and early referral or treatment at a dedicated transplant facility is recommended [13–15].

The differential diagnosis of ALI is broad and includes infection, congenital or genetic conditions, ischemia, and vascular and drug-induced etiologies [13]. An infectious workup should include testing for hepatitis B and C, CMV, EBV, and herpesvirus in immunocompromised patients [13, 14]. Congenital or genetic causes of preceding liver disease include Wilson's disease and hemochromatosis which can be evaluated by specialized genetic testing or through initial screening with serum ceruloplasmin and ferritin, respectively [11–13]. Ischemic liver injury produces profound and rapid elevations in ALT and AST due to hypoxic damage to hepatocytes [16]. ALI secondary to ischemia should be strongly considered in surgical patients, trauma patients, and patients with concomitant cardiac disease [16]. Drug-induced liver injury (DILI) is a diagnosis of exclusion and occurs following the introduction of a hepatotoxic medication or substance. In establishing a diagnosis of DILI, full consideration and evaluation of alternative diagnoses must be performed and there must be a temporal relationship between a medication or drug's introduction and the development of liver injury [14].

DILI may occur due to direct hepatotoxicity, indirect hepatotoxicity, or idiosyncratic drug reactions. While the pattern of liver injury and the onset of symptoms vary widely with the underlying mechanism of injury, pharmaceuticals with direct and indirect mechanisms of hepatotoxicity tend to be known precipitants of hepatic injury [14]. In contrast, idiosyncratic drug reactions tend to occur from medications with little to no known intrinsic hepatotoxicity and may be more difficult to identify as the cause of DILI [14]. As identifying the causative agent and discontinuing hepatotoxic medications are key components in DILI management, medications with unknown idiosyncratic drug reactions present a unique diagnostic challenge [15]. In this way, sevoflurane is generally considered to have minimal to negligible hepatotoxicity [2, 4]; however, multiple case reports have described rare instances of significant hepatotoxicity following sevoflurane use [5–7]. Generally, patients become symptomatic within the first three postoperative days following sevoflurane use with rapid elevations in ALT and AST consistent with a hepatocellular pattern of damage, new-onset mental status changes, and clinically apparent coagulopathy [5–7]. The outcomes of patients from DILI associated with sevoflurane have been mixed, with reported mortality [7–9], survival [6, 10], and significant morbidity, with one patient requiring liver transplantation [17]. Risk factors for developing DILI from sevoflurane include recent surgical procedures with sevoflurane anesthetic, previous exposure to sevoflurane, renal dysfunction, and immunocompromised status [6]. While the majority of cases described acute episodes of hepatic injury, one report described four patients with chronic hepatitis following sevoflurane use, indicating that the implications of sevoflurane hepatotoxicity are not limited to the immediate postoperative period [18]. Following the diagnosis of DILI from sevoflurane, patients should be cautioned to avoid further exposure to halogenated anesthetics as re-exposure may result in a similar or more severe reaction [4].

In our patient's case, the etiology of ALI was initially unclear. Autoimmune, infectious, ischemic, genetic, and drug-induced etiologies were evaluated through extensive laboratory workup which included autoantibody testing, viral panels, blood cultures, toxicology screening, and molecular testing. Following a nondiagnostic workup and evidence of a hepatocellular pattern of injury, ischemic and drug-induced hepatic failure were considered the most likely competing diagnoses. The patient's blood loss was minimal during the operation with stable blood pressure throughout the entirety of the procedure in addition to continuous intravenous fluid maintenance. Preceding the development of ALI, our patient received multiple agents with known or potential hepatotoxicity including methylprednisolone, mycophenolate mofetil, sevoflurane, and propofol. However, given that methylprednisolone, mycophenolate mofetil, and propofol were administered concurrently (immunosuppression and multiple O.R visits) for the duration of the hospital course as transaminitis resolved, these were considered less likely than sevoflurane as the causative agent. A dose or duration-dependent relationship could exist in sevoflurane hepatotoxicity especially in patients with concomitant kidney failure. Given the limitations of our study as a case report in establishing causation and evaluating sevoflurane as a definitive cause of our patient's ALI, we assessed our patient's reaction with the Roussel Uclaf Causality Assessment Method (RUCAM). This is a validated tool for assessing causality in liver toxicity from adverse drug reactions. Our patient's RUCAM score was 6, indicating a probable DILI [19].

The possibility of graft versus host disease (GVHD) was also discussed and was included as one of our differentials. However, the patient had no typical signs or symptoms suggestive of GVHD. The acuteness of clinical findings, absence of skin manifestations, fever, nausea, vomiting, and diarrhea pointed away from the diagnosis of GVHD [20]. A typical presentation for GVHD is 2–12 weeks after solid organ transplantation [20]. The assessment of macrochimerism (detection of > 1% donor nucleated cells in recipient peripheral blood film) was also negative in our patient.

## 4. Conclusion

We describe a case of ALI possibly secondary to sevoflurane exposure in a patient undergoing renal transplantation which ultimately led to the loss of the donor's kidney. The patient experienced significant morbidity resulting from ALI. Our study reinforces the importance of close postoperative monitoring of patients following kidney transplantation and adds to the current literature describing sevoflurane as a potential hepatotoxic agent. However, additional research is needed to elucidate the underlying mechanism of hepatotoxicity and identify those most at risk of developing DILI from sevoflurane. Although exceedingly rare, possible hepatotoxicity particularly in patients with renal dysfunction should be considered in the selection of anesthetic agents especially in solid organ transplant patients.

## Figures and Tables

**Table 1:** Table of laboratory values at key time points throughout the hospital course with listed reference values.

Laboratory test	Preop	POD 0	POD 1	POD 2	POD 12	Reference range
AST (U/L)	14	2673	4396	3851	36	11–35
ALT (U/L)	9	2680	1105	500	23	10–49
Alkaline phosphatase (U/L)	50	42	59	65	137	45–129
Total bilirubin (mg/dL)	0.7	0.8	2.0	2.1	0.7	0.3–1.2
Ammonia (umol/L)	—	137	64	69	—	10–50
Lactic acid (mmol/L)	—	10.0	5.6	2.2	—	0.5–2.2
INR	0.9	2.2	1.7	1.6	—	
PT (s)	10.6	25.4	19.0	18.2	—	10.1–12.9
PTT (s)	29.1	41.4	34.0	33.3	—	28.0–35.7

*Note:* Postoperative dating is relative to the initial kidney transplantation surgery.

## Data Availability

All data related to the study are available as an anonymous record.
